# An insight into the sialome of *Glossina morsitans morsitans*

**DOI:** 10.1186/1471-2164-11-213

**Published:** 2010-03-30

**Authors:** Juliana Alves-Silva, José MC Ribeiro, Jan Van Den Abbeele, Geoffrey Attardo, Zhengrong Hao, Lee R Haines, Marcelo B Soares, Matthew Berriman, Serap Aksoy, Michael J Lehane

**Affiliations:** 1 Vector Group, Liverpool School of Tropical Medicine, Liverpool, L3 5QA, UK; 2 Current address: The Healing Foundation Centre, Manchester, M13 9PT, UK; 3 Section of Vector Biology, Laboratory of Malaria and Vector Research, National Institute of Allergy and Infectious Diseases, Rockville MD 20852, USA; 4 Department of Parasitology, Unit of Entomology, Institute of Tropical Medicine, B-2000 Antwerp, Belgium; 5 Department of Epidemiology of Microbial Diseases, Yale School of Public Heath, New Haven, CT 06520, USA; 6 Children's Memorial Research Center, North-Western University, Chicago, IL 60614, USA; 7 Wellcome Trust Sanger Institute, Hinxton, CB10 1SA, UK

## Abstract

**Background:**

Blood feeding evolved independently in worms, arthropods and mammals. Among the adaptations to this peculiar diet, these animals developed an armament of salivary molecules that disarm their host's anti-bleeding defenses (hemostasis), inflammatory and immune reactions. Recent sialotranscriptome analyses (from the Greek *sialo *= saliva) of blood feeding insects and ticks have revealed that the saliva contains hundreds of polypeptides, many unique to their genus or family. Adult tsetse flies feed exclusively on vertebrate blood and are important vectors of human and animal diseases. Thus far, only limited information exists regarding the Glossina sialome, or any other fly belonging to the Hippoboscidae.

**Results:**

As part of the effort to sequence the genome of *Glossina morsitans morsitans*, several organ specific, high quality normalized cDNA libraries have been constructed, from which over 20,000 ESTs from an adult salivary gland library were sequenced. These ESTs have been assembled using previously described ESTs from the fat body and midgut libraries of the same fly, thus totaling 62,251 ESTs, which have been assembled into 16,743 clusters (8,506 of which had one or more EST from the salivary gland library). Coding sequences were obtained for 2,509 novel proteins, 1,792 of which had at least one EST expressed in the salivary glands. Despite library normalization, 59 transcripts were overrepresented in the salivary library indicating high levels of expression. This work presents a detailed analysis of the salivary protein families identified. Protein expression was confirmed by 2D gel electrophoresis, enzymatic digestion and mass spectrometry. Concurrently, an initial attempt to determine the immunogenic properties of selected salivary proteins was undertaken.

**Conclusions:**

The sialome of *G. m. morsitans *contains over 250 proteins that are possibly associated with blood feeding. This set includes alleles of previously described gene products, reveals new evidence that several salivary proteins are multigenic and identifies at least seven new polypeptide families unique to *Glossina*. Most of these proteins have no known function and thus, provide a discovery platform for the identification of novel pharmacologically active compounds, innovative vector-based vaccine targets, and immunological markers of vector exposure.

## Background

The superfamily Hippoboscoidea comprises higher flies (Suborder Brachycera Schizophora: Calyptrate), which includes the tsetse, louse flies, and the bird and bat flies [1]. These flies have in common the unusual ovoviviparous reproductive process. All adults are exclusively blood feeders on mammals or other vertebrates, suggesting that hematophagy is a monophyletic trait in this group [2]. The family Glossinidae has a single genus, *Glossina*, which comprises 31 species and sub-species of tsetse flies. Tsetse (which means *fly *in the south African Tswana language) are today found exclusively in sub-Saharan Africa and are of both medical and veterinary importance because they are vectors of African trypanosomes to humans and domesticated animals [3]. Surprisingly, blood is a very unbalanced meal, lacking many vitamins for example, and perhaps for this reason, tsetse flies have mutualistic endosymbionts that are required for successful fly reproduction, digestion and nutrition [4,5]. The intricate relationship between the parasites and the mutualistic endosymbionts indicate that the origin of blood feeding in this genus is ancient, probably during or before the mammal radiation of 60 million years ago (MYA). Indeed *Glossina *fossils from 38 MYA were found in the Florissant formation (Colorado), and also in Germany, indicating these flies were probably distributed worldwide 30-40 MYA [2].

Blood sucking arthropods must deal with their hosts' defense against blood loss (hemostasis based on blood clotting, platelet aggregation and vasoconstriction), as well as their defenses triggered by inflammation and immunity mechanisms. These defences may cause death to the insect or, at the least, interrupt bloodmeal acquistion. The saliva of haematophagous arthropods can counteract these barriers by using a complex mixture of pharmacologically active components, which are injected into the host skin during the probing and ingestion phases of feeding [6,7]. Accordingly, at the site of skin penetration, the hosts' response is pharmacologically modified by these salivary agents, which may inadvertently lead to enhanced transmission of pathogens. For this reason, the salivary contents of these vectors are not only a source of novel pharmaceuticals, but also can provide vaccine targets to interrupt disease transmission [8].

In the past 9 years, analysis of the salivary transcriptomes of bloodfeeding arthropods, including several genera within the ticks [9], triatomines [10-12], fleas [13], sand flies [8,14], *Culicoides *[15,16] and mosquitoes [17-21], have indicated that saliva contains a cocktail of 70 - 150 proteins (insects) to several hundreds of proteins (ticks, which feed for several days on their hosts). Because the evolution of blood feeding among insects occurred independently several times, the composition of the sialome differs substantially among insects not sharing a common blood feeding ancestor, thus representing a classical case of convergent evolution. However, variation among sialomes within the same family and even within the same genus is also observed [22], indicating the fast evolution of these proteins possibly due to host immune pressure. Indeed almost every genus of tick, mosquito or sand fly studied so far has novel proteins or protein families without similarity to any other known protein.

The non-redundant (NR) protein database of GenBank currently (Oct/2009) contains 156 proteins derived from the *Glossina *genus, 17 of which are annotated as found in the insect's salivary glands. These proteins were identified while characterizing a tsetse salivary anti-thrombin peptide [23], which molecularly defined some of the biological anti-haemostatic activities described before [24,25]. Salivary adenosine deaminases, antigen-5 related proteins [26,27] and endonucleases of the TSAL family have also been previously described. A limited transcriptome analysis of *Glossina morsitans *saliva identified eight additional proteins, including a proline rich protein, glycine-glutamate rich proteins, and a novel member of the TSAL endonuclease family [28]. In the present work, over 20,000 clones were sequenced from a normalized cDNA library from *G. morsitans morsitans *and analyzed in combination with previously described ESTs from adult tsetse fat body [29] and midgut [30], permitting a uniquely detailed analysis of the sialome of a haematophagous Dipteran.

## Results and Discussion

### cDNA library characteristics

A total of 22,794 clones from the salivary gland (SG) cDNA library of *G. m. morsitans *were assembled and merged, using a combination BLAST and CAP3 pipeline [31], with 19,998 previously reported ESTs from the fat body (FB) [29] and 19,459 ESTs from the midgut (MG) [30]. This EST assembly version dated 06/07/2007 was used in all analyses reported in the present study and comprises 62,251 ESTs assembled into 16,743 clusters (including 9,686 singletons), of which 8,506 had one or more sequences from the salivary gland cDNA library. A subsequent assembly version dated 12/17/2008 was produced by the International *Glossina *Genomics Initiative (IGGI) Consortium. Both versions are available from http://www.genedb.org. (Throughout the manuscript we will use the name contig or cluster to indicate both contigs and singletons).

Based on various BLAST sequence comparisons to several databases (see methods and Additional file 1), these clusters were functionally characterized in the following groups: Putative secreted (S), putative housekeeping (H), transposable element derived (TE), of viral (V) and microbial (M) origins, and of unknown class (U), because they could not be classified (some of which may be derived from untranslated regions of mRNA's (Table 1)). While most sialotranscriptomes completed to date have the larger set of ESTs attributed to the S class, it is possible that library normalization allowed for the H class to contain the larger number of ESTs (78.5% of the 22,794 ESTs) as well as of contigs (92% of the 8,506 contigs) (Table 1). Nonetheless, the S group still comprised 20% of the ESTs, and the ratio of ESTs per contig was the highest of all, with an average of eight ESTs per contig, biased by the high number of ESTs in particular salivary families, as will be indicated further below.

**Table 1:** Functional classification of transcripts from the salivary glands of *Glossina morsitans morsitans*

Class	Number of Contigs	Percent of contigs	Number of ESTs	Percent of ESTs	EST's/Contig
Housekeeping	5109	60.06	14427	63.29	2.82
Secreted	550	6.47	4582	20.10	8.33
Transposable element	95	1.12	162	0.71	1.71
Viral	12	0.14	29	0.13	2.42
Microbial	7	0.08	8	0.04	1.14
Unknown	2733	32.13	3586	15.73	1.31
					

Total	8506	100	22794	100	

The H class was further characterized (again based on similarities to various databases, in particular the KOG and Gene Ontology databases) into 21 functional groups (Table 2), the unknown conserved class being the most prevalent [32].

**Table 2:** Functional classification of the housekeeping transcripts from the salivary glands of *Glossina morsitans morsitans*

Class	Number of Contigs	Percent of contigs	Number of ESTs	Percent of ESTs	EST's/Contig
Unknown, conserved	1228	14.44	2702	11.85	2.20
Signal transduction	679	7.98	1839	8.07	2.71
Cytoskeletal	220	2.59	1031	4.52	4.69
Metabolism, energy	231	2.72	1019	4.47	4.41
Protein synthesis machinery	276	3.24	901	3.95	3.26
Protein export machinery	295	3.47	857	3.76	2.91
Transporters/storage	284	3.34	838	3.68	2.95
Protein modification machinery	259	3.04	825	3.62	3.19
Transcription machinery	321	3.77	804	3.53	2.50
Nuclear regulation	253	2.97	660	2.90	2.61
Metabolism, carbohydrate	164	1.93	571	2.51	3.48
Transcription factor	236	2.77	529	2.32	2.24
Proteasome machinery	157	1.85	455	2.00	2.90
Metabolism, lipid	172	2.02	454	1.99	2.64
Metabolism, amino acid	82	0.96	255	1.12	3.11
Metabolism, nucleotide	66	0.78	206	0.90	3.12
Oxidant metabolism/detoxication	59	0.69	172	0.75	2.92
Metabolism, intermediate	53	0.62	113	0.50	2.13
Extracellular matrix/cell adhesion	50	0.59	110	0.48	2.20
Immunity	9	0.11	44	0.19	4.89
Heme metabolism	15	0.18	42	0.18	2.80
					

Total	8506		22794		

Transposable element sequences are commonly found in sialotranscriptome. The sialotranscriptome of *G. m. morsitans *revealed both TE class I and class II transcripts, including mariner and piggyBac transposases and retrovirus sequences. These sequences may represent active transposition, or, more likely, the expression of regulatory sequences that might suppress the DNA transposition phenomena [33].

The few M class of transcripts found in the salivary glands were so classified due to their strong match to predicted bacterial proteins, including the genera *Neisseria *and *Rhizobium*. These clearly do not derive from the vector (*E. coli*), although they could be due to contamination or mislabeling of sequences, rather from being of symbiotic origin. A few transcripts also matched *Trypanosoma brucei*, as shown in additional file 1. These trypanosome sequences were found in the midgut EST library because parasite infected midgut material was used for the EST construction [29]. Finally the V class includes transcripts from the recently described *Glossina pallidipes *salivary gland hypertrophy virus [34-36], and also a polyprotein from a picorna-like virus, never described before and possibly specific for the salivary glands because all 17 transcripts were found only in the SG library.

The assembly of this multiple tissue EST set allows sequence frequency comparisons from different tissues by a Chi-square test. Accordingly, we found a total of 401 clusters that were significantly over or under expressed in one of the 3 libraries, 327 of which were associated with increased or decreased salivary gland transcript abundance. Of these 327 clusters, 59 were significantly over expressed, further implicating a specific salivary gland function (See worksheet named Sg-Upregulated in additional file 1), as will be described in more detail in the following sections.

The assembly of the three libraries also increased EST coverage for the gene products expressed on multiple tissues. We accordingly obtained 2,509 protein coding sequences with no full matches on GenBank, 2,279 of which are possibly full length proteins, the remaining being fragments of coding sequences that are considered of relevance to further studies. Of these deduced coding sequences, 1,792 were found expressed in the salivary glands by 1 or more EST, and include several that are mostly or solely found expressed in this secretory organ.

### Analysis of the Glossina *m. morsitans *sialotranscriptome

Several clusters of sequences coding for housekeeping and putative secreted polypeptides (indicated in additional file 1) are abundant and complete enough to extract consensus sequences that are typically absent from either GenBank or Swissprot. These sequences were grouped together in additional file 2, which also includes proteins previously described and deposited in GenBank. These are identifiable by their accession number (gi|), where the novel proteins have a GM prefix. The new coding sequences dicussed in this work were submitted to GenBank and have the accessions EZ421978-EZ424487. A detailed description of the sialotranscriptome of *G. m. morsitans *is provided to serve as a guide to browsing the two additional files.

### Possibly secreted (S) class of expressed genes

Inspection of additional file 1 indicates the expression of several gene families that encode secreted proteins, including endonucleases, exonucleases, 5'-nucleotidase/apyrases, adenosine-deaminases and mucins (Table 3), including familiar ubiquitous families of unknown function, such as antigen-5 and the yellow protein family. Protease inhibitors of the Kunitz and serpin families were also found, in addition to the previously described *Glossina *salivary anti-thrombin, and these may be associated with anti-clotting or anti-complement activities. Many of the transcripts reported are also linked to insect immunity and include serine proteases associated with prophenoloxidase activation cascades, as well as proteins related to pathogen recognition. Several other enzyme and peptide families, described in greater detail below, were also described.

**Table 3:** Classification of transcripts associated with blood feeding function

Class	Number of ESTs
Enzymes	
Nucleotide catabolism	
Endonuclease	3285
Rnase	19
Exonuclease 3'5' and 5'3'	28
5' nucleotidase/apyrase	44
Cimex-type apyrase (1)	2
Adenosine deaminase	46
Serine proteases (2)	54
Other proteases	10
Esterases and lipases	42
Lysosomal type phosphatase (1)	7
NO synthase	25
Oxidant metabolism	6
Possible PGE2 synthase	2
Hyaluronidase	2
Mucins	69
Antigen 5 family	66
Yellow protein family	5
Immunity related	
Alpha2 macroglobulin/TEP	15
Pattern-recognition	
Ficolins	197
C type lectins and galectins	49
Peptidoglycan binding protein	8
Defense response peptide	7
Similar to Drosophila virus induced peptide	13
Stomoxyn antimicrobial family	6
Protease inhibitor domains	
Anti thrombin peptide	1
Kunitz domain of serine protease inhibitor	1
Serpins	21
Low complexity families of secreted peptides	
Glycine rich family	24
Acidic proteins, probably secreted	40
Secreted proteins of conserved families	8
Secreted uncharacterized families	120
Possibly secreted proteins of unknown family (3)	360
	

Total	4582

(1) probably cellular

(2) possibly involved in phenol oxidase activation - may also be involved in cleavage of host proteins

(3) At least one orf has signal peptide - most probably are UTR's of mRNAs

Putative secreted salivary proteins containing ubiquitous domains, or ubiquitous protein families with or without known function:

#### Enzymes acting on nucleotides

Several transcripts found in the sialotranscriptome of *G. m. morsitans *encode proteins with sequence similarity to several secreted nucleotidases, ribonucleases, including endonucleases, exonucleases, 5'-nucleotidase/apyrases and adenosine deaminases, as follows:

##### Endonucleases

**Figure 1:**
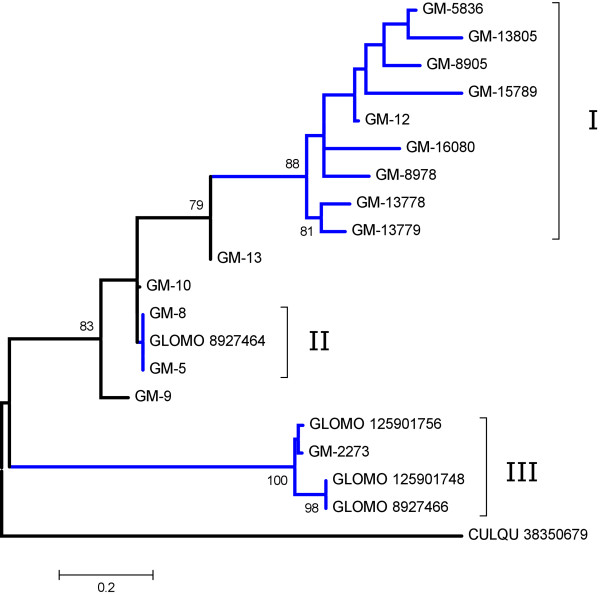
**Dendrogram of the Glossina morsitans morsitans salivary endonuclease-like proteins with the Culex quinquefasciatus salivary endonuclease included as an outgroup**. The *G. m. morsitans *sequences are indicated by GM-X where X is the number shown in additional file 2. The remaining sequences derived from the National Center for Biotechnology Information (NCBI) are represented by five letters followed by the NCBI gi| accession number. The five letters are taken from the first three letters of the genus and the first two letters from the species name. The protein sequences were aligned by the Clustal program [176], and the dendrogram was done with the Mega package [178] after 10,000 bootstraps with the neighbor joining (NJ) algorithm. Bootstrap values above 75% are shown in the nodes. The bar at the bottom represents 20% amino acid substitution. The roman numerals indicate clades discussed in the text.

Two putative proteins named Tsal 1 and Tsal 2 (this latter with two forms, A and B) were described in a previous sialotranscriptome of *G. m. morsitans *[27,28]. Not surprisingly, these genes were highly expressed in the salivary gland transcriptomes when compared to the two other tissue libraries. For example, in the salivary gland library, 2,874 EST's code for Tsal 1, but only 21 ESTs coding for this protein are found in the two other libraries. This library expression pattern is in accordance with previous work that determined Tsal 1 and Tsal 2 to be specifically expressed in the adult salivary glands by RT-PCR experiments [27]. These proteins had sequence similarity to endonucleases, but their function in tsetse saliva is unknown. These proteins were highly abundant (>40% of the total protein content) in *G. m. morsitans *saliva [28] and induced a strong humoral response in the mammalian host [37]. Sialotranscriptomes of sand flies have also revealed this family of proteins [14,38]. Recently, a salivary endonuclease of the mosquito *Culex quinquefasciatus *was cloned and the recombinant protein was expressed and shown to have activity toward double-stranded DNA. Strong salivary activity against the same substrate was also found [39]. It was postulated that this activity may decrease host tissue viscosity to facilitate diffusion of salivary pharmacological components through the dermis, and/or to produce small DNA fragments that have been demonstrated to have anti-hemostatic activity [40]. Additional file 2 shows 19 sequences with similarity to endonucleases (nine of which are full length) and includes the previously described proteins as well. Alignment of these sequences and phylogeny reconstruction (Figure 1) together suggest that these proteins resulted from gene duplication events leading to at least eight genes or more if there are genes coding for similar proteins. Accordingly, clade I in Figure 1 has strong bootstrap support for at least two related gene families, but includes in the same family individuals with more than 10% amino acid divergence, making it feasible that clade I is comprised of four genes. Clade II shows a possible polymorphic gene, with GM-8 and GM-9 being alleles of the previously described protein coded by gi|8927464. Clade III has possibly two closely related genes with two alleles each. Other genes may code for GM-9 and possibly GM-13, thus adding up to a minimum number of eight genes coding for this protein family. The abundance of these alleles may result from host immune pressure creating a scenario of balanced polymorphism and fast evolution. If this is the case, these proteins should be quite divergent in flies from the same genus and may represent good species markers, as has been demonstrated with the expanded family of triatomine lipocalins [41].

**Figure 2:**
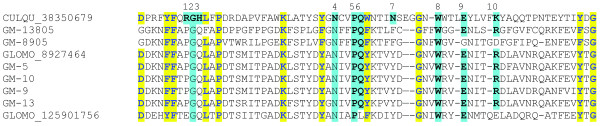
**Alignment of the active center region of Culex quinquefasciatus endonuclease with Glossina morsitans morsitans proteins of the same family**. The ten amino acids making contact to substrate, based on the *Serratia marcescens *crystal structure, are shown in turquoise background for the *Culex *and *Glossina *sequences. Note the absence of three conserved residues in the tsetse proteins. Other conserved residues are marked in yellow background.

The active center region of *Culex *endonuclease aligns well with bacterial and vertebrate endonucleases of the same family, including the enzyme from *Serratia marcescens *which has been crystallized, and the ten amino acids making substrate contact identified [42]. When compared to *S. marcescens *endonuclease, the *Culex *enzyme has conservation for all these amino acids (see Figure 1 in [39]). However, three of these substrate contacting amino acids are mutated in the *Glossina *proteins (Figure 2). Notably the substitutions are quite divergent (R -> P/E, H->Q and N ->F/Y), raising the doubt whether the tsetse proteins display endonuclease activity or whether this protein family has evolved to perform a different function. This anomaly has been detected previously in fleas, where an expansion of the acidic phosphatase protein family was identified in *Xenopsylla cheopis*. Members of the phosphatase family account for most of the salivary protein of this flea, but it has no phosphatase activity and all amino acids that should contact the substrate have been altered [13]. The role of the endonuclease protein family in *Glossina *remains to be identified.

##### Ribonucleases

Ribonuclease-like gene products containing a signal peptide were previously identified in the sialotranscriptomes of *Anopheles gambiae *[18] and *Aedes aegypti *[20], but neither their presence in saliva nor their function have been elucidated. Similarly, the salivary transcriptome of *G. m. morsitans *reveals two ribonuclease transcripts, one with a clear signal indicative of secretion (GM-1457), and the other with a borderline indication of secretion (GM-1723). GM-1457 belongs to the T2 family of ribonucleases, and is 60% identical to the RNaseX25 of *D. melanogaster *that belongs to a ubiquitous protein family [43]. This enzyme type was also reported in the sialotranscriptome of *Ae. aegypti *and the *Glossina *homolog is about 41% identical to the mosquito enzyme. Transcripts for GM-1475 were also found in the fat body and midgut, indicating ubiquitous tissue expression of this enzyme. The second ribonuclease, GM-1723 is similar to ribonucleases of *Ceratitis capitata*, which were shown to be a ubiquitous family [44] and was also found in the sialotranscriptome of the tick *Ixodes scapularis *[45]. Six transcripts were found for this enzyme in the salivary gland transcriptome, but none in the fat body or midgut libraries, suggesting an increased expression of this gene product in the salivary glands. Recently it was reported that a ribonuclease of the T2 family is a major component of *Schistosome *eggs responsible for conditioning dendritic cells for Th2 polarization [46].

##### Exonucleases

Four transcripts coding for 3'-5' exonucleases are shown in additional file 2, one of which has a clear signal peptide indicative of secretion. To the extent the salivary endonucleases are active, it is tempting to speculate that these transcripts could function in the further degradation of the endonuclease products. It is also possible that these exonucleases can hydrolyze diadenosine nucleotides such as Ap4A or Ap5A, which are pro-inflammatory purines released by platelets [47,48].

##### 5'-nucleotidases/apyrases

Enzymes that hydrolyze ATP or ADP to AMP and orthophosphate (ATP-diphosphohydrolase or apyrase) are ubiquitously found in the saliva and salivary gland homogenates of blood sucking insects and ticks [7], and were detected in *Glossina *salivary homogenates almost 30 years ago [24]. These enzymes are thought to increase the invertebrate fitness during feeding in two ways: first by decreasing local host hemostasis when ADP, released by damaged cells, induces platelet aggregation and second by decreasing neutrophil activation when ATP (also released by damaged cells and platelets) leads to neutrophil degranulation [6]. Indeed *Glossina *salivary homogenates inhibit ADP-induced platelet aggregation *in vitro *[24].

At least three gene families have been proposed to fulfill the apyrase task among different arthropods. Bed bugs and sand flies have opted for the Ca^++ ^dependent *Cimex *family of apyrases [49,50], mosquitoes and kissing bugs of the genus *Triatoma *for the 5'-nucleotidase family [51-53], and fleas may possibly use the CD-39 gene family [13]. The *G. m. morsitans *sialotranscriptome indicates the presence of at least four different transcripts coming from possibly three different genes that belong to the 5' nucleotidase gene family. These transcripts match NCBI deposited proteins from *G. m. morsitans *annotated as salivary 5' nucleotidase from an unpublished work (gi|14488055 and gi|126143295). GM-784 is 99% identical to gi|14488055 and is probably an allele. GM-541 is a truncated transcript identical to gi|126143295, but GM-541 is only 89% identical to gi|126143295 and 68% identical to gi|14488055 and may be derived from a different gene. The protein coded by gi|14488055 has EST expression in all three libraries (38 ESTs), however it has increased representation in the salivary gland library (25 ESTs). On the other hand, GM-541 has ten ESTs in the salivary gland library and none in the other two libraries, indicating a possible increased salivary expression of this gene product. These 5' nucleotidase sequences match the haematophagous horse fly salivary protein named chrysoptin precursor, which has been described as an inhibitor of collagen induced platelet aggregation [54], a function that would be performed by apyrases.

**Figure 3:**
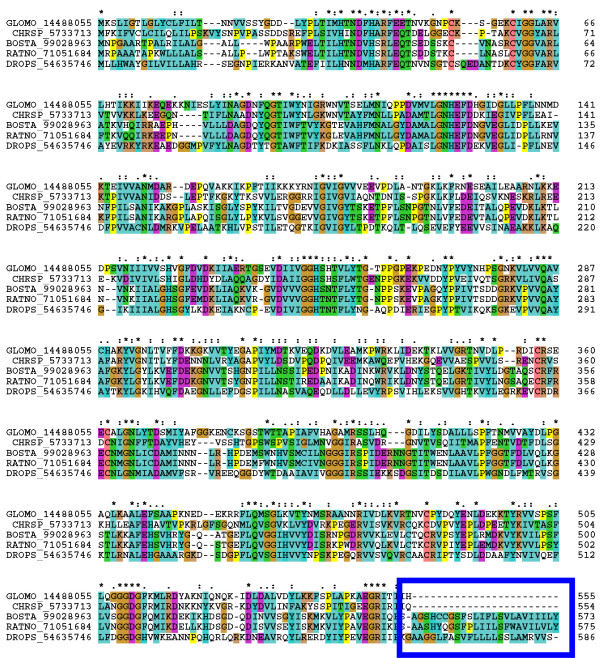
**Alignment of members of the 5' nucleotidase family from D. pseudoobscura, Bos taurus, Rattus rattus, Glossina morsitans morsitans and the horse fly Chrysops spp**. The numbers following the species abbreviations indicate the NCBI accession number for each protein. Notice, in the haematophagous dipteran sequences, the absence of the carboxyterminal region where the glycophophatidylinositol anchor normally attaches (indicated by the blue box). Symbols above the alignment indicate standard ClustalW nomenclature: (*) identity, (:) high conservation and (.) conservation.

5' Nucleotidases are typically extracellular proteins bound to the membrane by glycosylphosphatidylinositol (GPI) anchors attached to their carboxyterminal domain. However, secreted 5'nucleotidase/apyrases lack the amino acids needed for anchor attachment [20,21,38,52], either through mutation or truncation. Alignment of bovine, rat, *Drosophila*, *Glossina *and tabanid sequences (Figure 3) clearly shows that the sequences derived from the bloodfeeding Diptera lack the GPI anchor attachment domain, thus inferring that these proteins are secreted. Whether a member of the 5' nucleotidase performs the apyrase function in *Glossina *remains to be determined.

In relation to apyrase, a homolog for the *Cimex *apyrase was also found in the *Glossina *transcriptome, indicated by one EST each from the salivary gland and from the midgut libraries. Although this enzyme was first described in *Cimex *salivary glands [49], it was later found to be part of a ubiquitous protein family including intracellular enzymes [55]. GM-8153 is truncated in its 5' region and accordingly it cannot be determined if the protein would be secreted or not. However, the *D. melanogaster *homolog does not have a signal peptide indicative of secretion, nor do the homologs of *Ae. aegypti *or *An. gambiae*, indicating that this apyrase is an intracellular enzyme from a conserved apyrase subfamily. Because the *Cimex *type enzyme is strictly Ca^++ ^dependent, evaluation of the divalent cation dependence of the salivary apyrase of *Glossina *could help to sort out the apyrase family. The work of Mant and Parker in 1981 demonstrated apyrase activity only in the presence of calcium ions [24], thus not allowing distinction between apyrase family types. If the salivary apyrase of *Glossina *works with Mg^++ ^in the presence of EGTA, the *Cimex *type of apyrase could be excluded from contributing to the activity.

##### Adenosine deaminase

Expression of genes coding for salivary adenosine deaminases (ADA) have been described previously in *G. m. morsitans *[27] and have been a common finding in the sialotranscriptomes of blood sucking Diptera, including sand flies [56,57] and culicine (but not anopheline) mosquitoes [58]. Additional file 1 presents eight protein sequences that belong to the ADA family, including the previously described gi|5817644 and one possible allele coded by GM-1228. This gene is abundantly expressed (27 ESTs) but not exclusively in the salivary glands, where ten ESTs were found. GM-536 codes for a novel adenosine deaminase having 54% identity to the *D. melanogaster *homolog, having 16 salivary ESTs from 21 total. GM-5870 and GM-537 are putative full length, but shorter versions of ADA enzymes with four ESTs each in the salivary library and none in the others. Finally, GM-547 and GM-2098 code for truncated transcripts of the ADA family, with only one, and two ESTs exclusively found in the salivary gland library. Combined, these results indicate that at least seven genes code for salivary gland ADA enzymes, some of which may be uniquely expressed in this tissue. It has been proposed that the function of this enzyme activity in haematophagous saliva may be to convert adenosine, a pain inducer and mast cell degranulating agonist, into inosine, that has > ten fold less degranulating activity [58].

##### Other enzymes

###### Hyaluronidase

Salivary hyaluronidase has been described before in the sialotranscriptomes of *Culex quinquefasciatus *and sand flies [21,38]. In addition, the activity has been demonstrated in the salivary glands of sand flies and black flies [59,60], where it may be adaptive to the insect by increasing the spread of pharmacological agents into the host skin, analogous to the "spreading factor" of bacteria that was later shown to be hyaluronidase [61]. Contigs 4069 and 4070 in additional file 1 represent partial sequences of hyaluronidases that might assist in such a salivary function if they are secreted in saliva.

###### Phospholipases

The sialotranscriptome of *G. m. morsitans *indicates expression of several esterases and phospholipases possessing a signal peptide indicative of secretion. One of these is similar to secreted phospholipase A2 (PLA2) and the other two are similar to lysophospholipases. To the extent that these enzymes are secreted in the saliva, they might produce pharmacologically active lipids in the site of the feeding, or help to hemolyse the erythrocytes after ingestion. Phospholipases have been found before in haematophagous arthropod sialotranscriptomes. PLA2 activity was previously demonstrated in tick saliva [62,63], and a phospholipase C activity found in *Culex quinquefasciatus *saliva and salivary gland homogenates destroys the lipid platelet aggregation agonist PAF (Platelet activating factor) [64].

###### Nitric Oxide synthase

**Figure 4:**
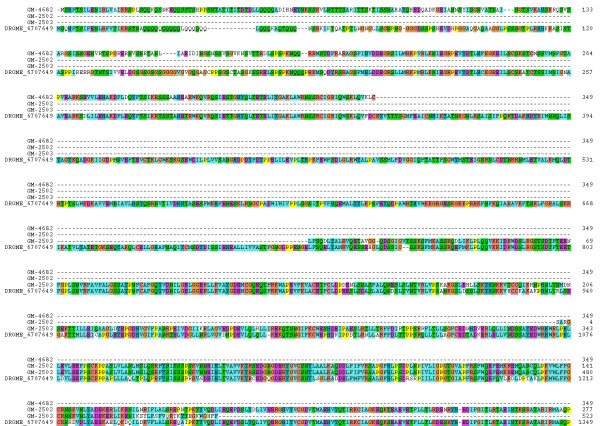
**Alignment of Drosophila melanogaster NOS protein (gi|6707649) with three deduced fragments of the NOS from Glossina morsitans morsitans**. The *G. m. morsitans *sequences are indicated by GM-X where X is the number shown in additional file 2.

**Figure 5:**
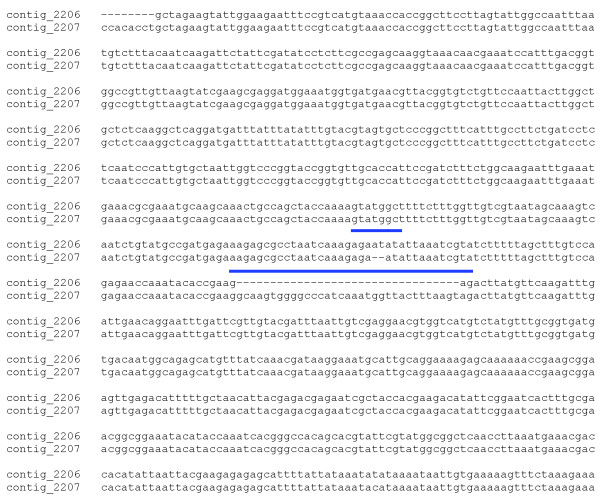
**Differential splicing of the nitric oxide synthase gene product in the salivary glands of *Glossina morsitans morsitans***. The blue lines are above regions of differential splicing.

A total of 22 ESTs were found exclusively in the salivary gland library coding for different portions of nitric oxide synthase (NOS). Three ESTs were also found encoding NOS interacting protein, with this last cluster also having two ESTs discovered in the fat body library (additional file 1). These transcripts either indicate the salivary glands utilize NOS as a signaling molecule, or that NOS or a NOS-adduct may be secreted in the saliva to produce anti-platelet and vasodilatory activities, as is the case with the hemipterans *Rhodnius prolixus *[65] and *Cimex lectularius *[66]. Of the three EST clusters coding for NOS, one codes for the amino terminal portion and the other two for overlapping regions of the carboxy terminus (Figure 4). The reason these last two clusters did not assemble together is due to differential splicing of the NOS message (Figure 5). The NOS gene in vertebrates is known to generate many alternative splice variants with importance to differential tissue expression, enzyme activity [67] and even, erection of the penis [68]. Further investigation of the NOS isoforms in the salivary glands of *Glossina *is warranted. GM-5084 represents the protein sequence of the *Glossina *homologue of the nitric oxide synthase interacting protein (NOSIP), which is an endogenous inhibitor of the enzyme important for the regulation of NO output in neuronal cells [69].

###### Prostaglandin E2 synthase

Two ESTs from the salivary gland library, together with three from the fat body library assembled to provide for the protein sequence named GM-4956, which has the CDD domains GST_C_mPGES and GST_N_mPGES2 indicative of the microsomal prostaglandin E synthase Type 2 (mPGES2) subfamily. This enzyme may produce an endogenous mediator of salivary gland function, or may indicate that tsetse saliva may contain PGE_2_, which is a potent skin vasodilator [70]. PGE_2 _and other lipid mediators have been found in the saliva of ticks [71-75], but so far never characterized in insect saliva.

###### Serine Proteases

Trypsin-like serine proteases play a role in digestion, where relatively small enzymes (about 220 aa in length) are found that lack substrate specificity. Larger enzymes function in more specific ways, for example, in the activation of proteolytic cascades of immunity pathways (such as activating the prophenoloxidase enzyme that produces pathogen melanization) [76,77]. These larger enzymes have additional domains that confer unique specificity to substrate proteins that will fit into the catalytic groove of the enzyme, or associate the enzyme in multimeric complexes [78,79]. Thirty three clusters, with a total of 54 ESTs from the salivary gland library code for proteins of the serine protease family with at least one EST present in the salivary gland library (Additional file 1). Additional file 2 provides for the full as well truncated sequence of 25 such enzymes, which includes two sequences previously described during the analysis of the *G. m. morsitans *fat body library [29], as well as the analysis of the proventriculus cDNA library [80]. It is possible that the majority of these enzymes function in insect immune pathways, which is indicated by their size and ubiquitous library expression. However, GM-5815 codes for a serine protease of 406 aa with 50% identity to a *Drosophila *enzyme. Four ESTs were detected in the salivary gland library but no other library. Similarly, 11 other sequences coding for serine proteases were found exclusively in the salivary gland library. To the extent that some of these proteins are secreted in saliva, they could act as plasminogen activators, on fibrinolysis or protein C activation. However, previous work with *G. m. morsitans *salivary gland homogenates failed to detect plasminogen activators or fibrinolytic enzymes [25]. It remains to be verified whether any trypsin-like activity is released into *Glossina *saliva.

###### Phosphatases and collagenase

Additional file 2 provides for one full and one partial sequence coding for a phosphatase of the acidic/lysosomal type as indicated by the KOG database. These enzymes indeed may be lysosomal, but are highlighted here because fleas have co- opted this gene family to be abundantly expressed in their salivary glands. GM-6557 codes for a fragment matching a KOG entry indicative of collagenase-related metalloprotease, which could play a role in blood feeding, if secreted.

##### Protease inhibitors

###### Anti-thrombin peptide

The salivary anti-clotting of *G. m. morsitans *was previously identified as an anti-thrombin [25], and was later molecularly characterized as a small (53 aa) and unique peptide [23]. Only one EST for this peptide was found in the salivary gland cDNA library, despite the abundance of this product in the salivary glands of adult *Glossina*. This poor representation may be the result of the library normalization protocol and the removal of small transcripts, pointing to the possibility that small transcripts coding for peptides may be lost when this library design is used.

###### Serpins

Additional file 2 also presents sequence for five members of the serpin (**ser**ine **p**rotease **in**hibitor) family of proteins that were found expressed in the salivary glands (all of which have been described in previous work analyzing the fat body transcriptome of *G. m. morsitans *[29]). The ubiquitous tissue expression of these serpins indicates they may function primarily in the control of proteolytic cascades such as in prophenoloxidase activation [81]. Notwithstanding this, a serpin family member contributes to the anti-clotting activity of *Aedes aegypti *saliva, which specifically blocks Factor Xa of the blood coagulation cascade [82].

###### Kunitz domain peptide

The Kunitz domain was first identified in serine protease inhibitors of vertebrates, such as the bovine pulmonary trypsin inhibitor (BPTI) and found later to be ubiquitously present in animals and plants. Potent inhibitors of the clotting cascade are found in proteins containing two or more such domains, each one interacting with a different protease of the hydrolytic cascade, as found in tick saliva [83,84]. Single Kunitz peptides also exist, and these may display additional activities such as antimicrobial [85], channel blocking, as recently characterized for a tick salivary protein [86], or inhibitors of other enzymes [87]. The protein sequence named GM-16650, derived from a single EST found in the SG library, codes for a single Kunitz peptide with signal sequence indicative of secretion, whose function remains to be identified.

###### Small molecule binding families

The secreted small protein coded by GM-1704 is a member of the insect pheromone binding family as indicated by its PFAM match. It is also similar to an ejaculatory bulb protein from *Drosophila *as indicated by a SwissProt match. Similarly, GM-4458 codes for a member of the phosphatidylethanolamine binding protein. ESTs from all three libraries are represented in both assemblies, indicating these proteins are not salivary gland specific. It should be remarked here that proteins of the odorant binding family are abundantly expressed in the salivary glands of haematophagous Nematocera (the so called D7 protein family), and lipocalins in ticks and triatomines bugs, where they can constitute a high proportion of the ESTs in sialotranscriptomes [6]. No transcripts coding for these protein families were found in the *Glossina *salivary gland cDNA library.

#### Ubiquitous annotated protein families, function unknown or unclear

##### Antigen 5 (AG5) family

AG5-related proteins belong to an ubiquitous group of secreted proteins within the CAP family (**c**ysteine-rich secretory proteins; **A**G5 proteins of insects; **p**athogenesis-related protein 1 of plants) [88]. Most of these animal proteins have no known function; in the few instances to the contrary, their function diverges from proteolytic activity in *Conus *[89], to smooth muscle-relaxing activity in snake venoms [90,91], to salivary neurotoxin in the venomous lizard *Heloderma horridum *[92]. Members of the AG5 family are found expressed in the salivary glands of all bloodfeeding insects studied so far [14,16,93]. In mosquito salivary glands, several genes of this family are expressed, some of which are salivary gland specific [17,18]. One member of this gene family (gi|8927462) was previously described in a transcriptome analysis of *G. m. morsitans *salivary glands [27]. This gene appears to be ubiquitously expressed, with 11 ESTs from the fat body, 25 from the salivary glands and 16 from the midgut libraries. We presently describe three additional protein sequences of this family, all of which are exclusively found in the salivary gland library, including two which had 18 ESTs each, and no ESTs in any of the other two libraries, a significant departure from expected equal distribution among the three libraries. GM-486 appears to be a differentially spliced form of the previously described protein, while GM-1862 and GM-485 are 80% and 93% identical to gi|8927462, respectively. Recently, the immunogenicity of members of this family in *Glossina *has been reported [94]. In the stable fly *Stomoxys calcitrans *a member of the AG5 family was shown to be abundantly expressed and to specifically bind immunoglobulins [95], perhaps having an anticomplement activity. Exceptionally, a tabanid salivary protein of this family incorporated an RGD domain [96-98] and became a potent platelet aggregation inhibitor [99].

##### Yellow protein family

Disruption of the *yellow *gene in *Drosophila *produced a yellow phenotype indicating it could mediate a melanization function. It was later shown that it coded for a dopachrome isomerase, which is important in the melanin formation pathway. Several genes of the yellow family exist in *Drosophila*, but not all display the isomerase activity [100]. Sand flies (but not mosquitoes) have abundant salivary expression of this class of genes [14,38], but no dopachrome isomerase activity was found in the gland (Ribeiro, unpublished). It is possible that this protein exercises binding of biogenic amines such as displayed by histamine/serotonin/norepinephrin binding proteins found in the salivary glands of ticks [101], mosquitoes [102] and triatomine bugs [103,104]. Incidentally, the major royal jelly protein of bees is related to the yellow family of proteins. The sequences of GM-4104 and GM-2745 code for two diverse members of this protein family that are expressed in the salivary glands, fat body and midgut of *G. m. morsitans*. Both have the PFAM domain MRJP that indicates this protein family (Additional file 2). They are 61 and 57% identical to the yellow-c and yellow-f2 proteins of *Drosophila*, respectively. The ubiquitous tissue expression of these two proteins is suggestive of a housekeeping role for this protein in *Glossina*.

##### Mucins

Under this class we include diverse serine + threonine rich secreted proteins that have in common a large number (> ten) of potential O-N-acetylgalactosylation sites as identified by the NetOGlyc server [105] and can thus be categorized as mucins. Such proteins have been regularly found in sialotranscriptomes of insects and ticks where they are postulated to help maintain the insect mouthparts, in addition to other possible functions. Nine such proteins are described in additional file 2, including a member of the ubiquitous hemomucin family [106,107], which was previously described in the fat body transcriptome of *G. m. morsitans *[29]. GM-2799 is a truncated protein sequence homologous to a *D. melanogaster *protein that contains a signal sequence, but the function remains unknown. GM-3365 has a signal sequence indicative of secretion, 23 putative glycosylation sites, and is also homologous to a *D. melanogaster *protein of unknown function. GM-1194 and GM-2819 are also similar to *D. melanogaster *proteins of unknown function. They have 12 and 15 glycosylation sites, respectively. GM-5570 represents a partial sequence for a mucin containing at least 40 glycosylation sites. The pair GM-1014 and GMN-14512 are related, this last sequence being a truncated relative. Each has 40 predicted glycosylation sites. Finally, GMN-15884 codes for a secreted protein with 15 glycosylation sites and no great similarities to any know protein. Most of these mucins are not represented in the Sanger assembly possibly due to their masking of low complexity regions.

##### Pathogen recognition proteins and defense response

It has become apparent from previous sialotranscriptomes that the saliva from haematophagous arthropods contains antimicrobial compounds, such as lysozyme, defensins and cecropins, as well as many peptides of unknown function that may be novel antimicrobial compounds. Some of these previously identified proteins are initiators of innate immune response cascades such as the phenol oxidase cascade described above in the serine protease section. It is probable that such compounds are secreted in saliva, where they either kill or opsonize the pathogens thus preventing the ingested blood from becoming a microbial haemoculture. Remarkably, the sialotranscriptome of *G. m. morsitans *analyzed in this work lacks some of the smaller peptides found in other sialotranscriptome, yet it abounds in other protein families associated with pathogen recognition, as shown below. This is probably due to the loss of small transcripts during the library normalisation process.

###### Lectins

Lectins are proteins with carbohydrate recognition domains associated with innate immunity [76] and intracellular Golgi protein transport [108]. C-type lectins found in snake venoms can display anti-platelet and anti-clotting activities [109,110]. The sialotranscriptome of *G. m. morsitans *had many clusters of ESTs producing matches to lectins, including several lectins previously described from the analysis of the *Glossina *fat body library. Specific to the salivary gland transcriptome, however, 49 ESTs assembled into 11 contigs; two of which are significantly up regulated in the salivary glands and contain either 18 or 13 transcripts (and zero transcripts in the other two libraries). The protein sequence of one of these transcripts is represented by GM-591, possibly coding for a galactose-specific C-type lectin, and similar to salivary proteins found in *Ae. aegypti *and *Ae. albopictus*. Contig 491, with 13 ESTs, may represent a chimeric assembly. A protein sequence was not deduced for this contig, but can be inspected in additional file 1. The sequence GM-595 codes for a short lectin that is not closely related to other family members. The functions of these putative salivary specific proteins remain unresolved.

##### Peptidoglycan recognition protein

Previous analysis of the *Glossina *fat body transcriptome uncovered the sequence of a pathogen recognition protein implicated in the initiation of innate defense mechanisms [29]. Three ESTs for this protein are represented in the salivary gland library. Additional file 2 describes a second member of this family, with 4 salivary ESTs and one each in the other 2 libraries. GM-4559 is closely related to a *Drosophila *protein and contains a KOG LysM domain indicative of peptidoglycan recognition.

##### Fibrinogen-domain-containing/Ficolin proteins

This group of proteins has the PFAM fibrinogen C motif and the KOG Ficolin motif seen in invertebrate proteins displaying lectin activity toward N-acetylglucosamine residues and implicated in immune function [111-113]. The PFAM motif is promiscuous, appearing in many other proteins such as vertebrate fibrinogens, angiopoietins and ficolins. Salivary glands of *Aedes *mosquitoes abundantly express this family of proteins, two of which were selectively expressed in the salivary glands of *Ae. aegypti *[20] and *Ae. albopictus *[17]. The sialotranscriptome of *Glossina *revealed abundant transcription of this gene family, producing 197 ESTs in nine clusters, with only 16 other ESTs deriving from the midgut but none from the fat body library. Notably, two clusters had 93 and 83 transcripts and were significantly more expressed in the salivary glands than other organs (additional file 1). Additional file 2 provides sequence information for six members of this family, four of which are full length, including the two abundant salivary transcripts. Closer inspection of this family of proteins indicates they belong to the ficolin subfamily as indicated by their KOG match. Notice that while the fibrinogen C motif is to only a portion of the sequence, the ficolin match is to the whole extent of these proteins that are usually 350 aa in length. Ficolins are lectins playing a role in innate immunity in both vertebrates and invertebrates [111,112]. These six protein sequences appear to derive from at least three different genes. GM-312, GM-313, GM-314 and GM-315 are related and could originate from alleles or from closely related genes. The remaining two proteins are more than 10% different from each other and may derive from two additional genes. These proteins may have been co-opted to play a role in neutralizing host hemostasis or inflammation instead of performing an immune function.

##### Thioester containing protein

Previous analysis of the fat body transcriptome of *G. m. morsitans *uncovered a protein containing similarity to alpha-2 macroglobulin, which has a reactive cysteine that can form a thioester bond to other, usually pathogen, molecules [114], in a manner similar to the vertebrate complement system. These thioester proteins (TEP) are known to be important in mosquito and *Drosophila *immunity [115,116]. In addition to fat body expression, six transcripts coding for this protein were also found in the salivary gland library.

##### Defense response associated proteins

Although of unclear function or mode of action, three protein sequences are presented in additional file 2 and are similar to *Drosophila *proteins associated with defense response. GM-431 and GM-434, probably allelic, code for secreted proteins of 22 kDa. The *Drosophila *homolog named vir-1-PC is virus induced. Similarly, GM-936 is similar to a Cys rich protein from *Drosophila*, which is annotated at Flybase as participating in the defense response.

##### Secreted polypeptides of conserved proteins of non-described families

Four full length and four carboxy truncated polypeptides have significant matches to *Drosophila *or other insect proteins of unknown function. All have a signal peptide indicative of secretion. GMN-2518 matches the *Drosophila lethal *gene product which is described in FlyBase as having unknown function. It has a PROSITE match to the Lipoyl pattern (2-oxo acid dehydrogenases acyltransferase component lipoyl binding site - [GDN]-x(2)- [LIVF]-x(3)-{VH}-{M}- [LIVMFCA]-x(2)- [LIVMFA]-{LDFY}-{KPE}-x-K- [GSTAIVW]- [STAIVQDN]-x(2)- [LIVMFS]-x(5)- [GCN]-x- [LIVMFY].) and may have a housekeeping function. No other insights were obtained for the remaining seven conserved polypeptides.

##### Possibly multi genic Glossina-specific salivary secreted polypeptides

Previous sialotranscriptomes of mosquitoes, triatomine bugs, sand flies and ticks consistently revealed genus-specific proteins (e.g. proteins that produce no similarity matches to known proteins) that are, in many cases, represented by multi-gene families. These novel families might have been derived from known genes that were altered by rapid evolution, perhaps as a consequence of immune pressure exerted by the vertebrate host. They may also represent unique natural experiments on exon shuffling resulting from the organism's genome scramble to obtain more blood from their hosts.

##### GE rich salivary proteins

Twenty six ESTs found exclusively in the salivary gland library assembled into five different contigs from which five protein sequences were derived, four of which are full length having a signal peptide indicative of secretion. One of these five proteins, GM-3689, is 100% identical to *G. m. morsitans *proline rich protein (gi|126143293), while the remaining proteins are 85-96% identical to *G. m. morsitans *glycine/glutamate-rich proteins (gi|126143291) [28]. Whereas GM-2815 and GMN-14464 may be alleles, the remaining proteins probably originate from different genes. These proteins are weakly similar to the 30 kDa family of proteins found to be exclusively expressed in the salivary gland of adult female mosquitoes, [17,18,20], but only because some of the *Glossina *proteins have multiple dipeptide (Gly-Glu) repeats that are part of a domain in the 30 kDa mosquito family. The mosquito proteins have a predicted molecular mass varying from 18-29 kDa, while the *Glossina *proteins are only half as large, ranging from 7.5-14.3 kDa. These proteins may have stemmed from a common ancestor and subsequently evolved to maintain only their scrambled Gly-Glu repeats, or they may be the product of convergent evolution. The role of any of these protein members in *Glossina *remains to be elucidated; in mosquitoes, the 30 kDa family has been characterized as inhibitors of collagen-induced platelet aggregation [117,118].

##### Glycine-Proline rich family

Nineteen ESTs derived from the salivary gland library plus three ESTs derived from the other two libraries assembled into three contigs coding for one protein containing a signal peptide and two other related protein fragments. The best similarity matches for this protein group are to hypothetical protozoan and plant proteins from *Paramecium*, *Trichomonas *and *Oriza*, deduced primarily by the regularly spaced residues, Pro, Asn and Tyr, which are also found in some transcription factors. This protein family is not available in GeneDB due to their masking of low complexity transcripts.

##### Fat body and salivary 20 kDa family

Twenty two ESTs derived from the fat body library plus 11 from the salivary gland library assembled to produce five related proteins that have no significant similarity matches to other known proteins. While GM-2258 and GM-2259 may be alleles, the other three proteins are more distantly related, indicating that at least four genes exist for this novel protein family. Members of this *Glossina *family all have a signal peptide, a mature molecular mass that varies from 18.9-20.2 kDa and a slightly acidic pI varying from 5.6-6.0. They are named FB-SG-20 Kda family. Because unique salivary protein families specific to disrupting host hemostasis or inflammation tend to be specifically expressed in the salivary glands, the discovery of this unique family expressed in the fat body suggests an antimicrobial function (although it may simply play a housekeeping role).

##### Salivary acidic 8 kDa peptide family

Additional file 2 contains the sequence information for three acidic (pI 3.8-3.9) polypeptides of predicted mature mass of 8.2-8.9 kDa that are likely to be the product of several gene duplication events. GM-1774 and GM-1775 appear to be splice variants, and GM-1937 a more distant relative. No significant matches are found for any member of the family when they are compared to the NR database. These three polypeptides are represented by a total of 18 ESTs found exclusively in the salivary gland library, with GM-1774 having statistically significant score, suggesting a specific function in this tissue. Notably, they have a carboxy terminus rich in Asn and Gln.

##### Basic 6.5 kDa family

Two distantly related basic peptides are coded by GMN-129 and GMN-4307. A total of nine ESTs from the salivary (five) and midgut (four) libraries were used to assemble these two peptides. They have an unusual conserved Phe rich character determined by the pattern block F-x(5)-S-F-x(2)-F-x(11,12)-L-S-x(4)-F-F-F- [FI]-I- [FY].

##### Basic 6 kDa family

GMN-16121 and GMN-6827 code for two related peptides that share a relatively conserved signal peptide, and a predicted Phe-Tyr rich mature polypeptide. They have no significant sequence similarities in available databases.

##### 5.8 kDa histidine rich peptide family

Two histidine-rich related secreted peptides with a mature molecular mass of 5.8-5.9 kDa correspond to two ESTs from the salivary gland library. GMN-14221 has Thr- His- repeats that produce matches to much larger hypothetical proteins found in the NR database, and may be related to transposons. The repeated histidine pattern may indicate metal binding domains that are common in Zn binding peptides that have antimicrobial activity [119,120]. Histidine repeat peptides have been commonly found in sialotranscriptomes. Whether they derive from a common ancestral gene that mutated fast is not known.

##### Basic 4 kDa family

Another two related secreted peptides, sharing a basic pI and predicted mass near four kDa, were deduced from three ESTs found in the salivary gland library.

##### Basic 3 kDa family

Two distantly related basic peptides are coded by GMN-122 and GMN-7766. A total of five ESTs from the salivary and midgut libraries were used to assemble these two peptides. They have a conserve Leu-Asn-Asn-Asn tail.

##### Other glutamate rich proteins

Four proteins rich in glutamate, two of them possibly the result of alternative splicing, have repeated glutamates and for this reason gives relatively high scores to other repeat rich proteins.

##### Other possibly salivary secreted polypeptides

Additional file 2 lists an additional 129 potentially secreted polypeptides with no significant matches to other known proteins, or matches to proteins of unknown function. It is possible that some (or many) of these hypothetical peptides are derived from truncated coding sequences and are thus only the 3' remnants that may contain a spurious signal peptide after a Met codon is found, as is the case in many transporters. Because we want to avoid excluding possible novel peptides in our analysis, we included them here despite the risk that we may incorporate erroneously predicted secreted peptides into our analyses. A few of the peptides in this category appear to be salivary gland specific, as they contain six or more ESTs found only in the salivary gland library. These include GM-2233, which actually has a match to a *Drosophila *protein, but this is predominantly due to the conserved Cys framework that matches the PROSITE EGF_2 pattern (C-x-C-x(2)- [GP]- [FYW]-x(4,8)-C.). Twelve transcripts coding for this protein were found solely in the salivary gland library. Seven ESTs, also exclusive of the salivary glands, code for GMN-3996, a small (53 aa) secreted polypeptide with a mature molecular mass of three kDa. Seven transcripts also assembled to provide for the protein sequence of GM-4306, which codes for a mature peptide of 7.8 kDa with a very basic pI (11.6). Two other unique peptides are coded by the sequences GM-1770 and GMN-2557, both with six ESTs exclusive of the salivary glands. GM-1770 has a lysine rich basic tail that is similarly found in an expanded protein family found in the salivary gland of ticks [45,121] and culicine mosquitoes [17,20]. These polyK tails may lead the peptide to interact with charged phospholipids such as those important for assembly of clotting cascades [122]. Indeed, one tick salivary anti-clotting protein named Salp14 is a member of the basic tail family [123].

### Housekeeping (H) genes

The 5,089 clusters (comprising 14,392 ESTs) recognized as H genes were further characterized into 24 subgroups according to function (Table 2). While previously reported sialotranscriptomes, which use non-normalized libraries, showed transcripts coding for protein synthesis machinery and energy metabolism as the most abundant, the top three classes of transcripts in the *G. m. morsitans *sialotranscriptome were for conserved proteins of unknown function, signal transduction and cytoskeletal proteins. Proteins associated with energy metabolism and protein synthesis followed these three groups. This difference from previous transcriptomes certainly relies on the library normalization protocol used here but not in previous work, allowing an unprecedented coverage of lower abundance transcripts. The complete list of all 5,089 gene clusters, along with further information about each, is given in additional file 1, and many of the annotated CDS are found in additional file 2. Below we highlight some transcripts from the H group that might be relevant to specific salivary gland function:

#### Nuclear regulation (mitosis, DNA repair and other nuclear maintenance)

Two hundred and fifty three clusters contain one or more EST expressed in the SG library under this class, 68 of these are shown in additional file 2 as CDS, mostly full length. This includes a histone tail methylase, as predicted by the KOG database, which has 20 ESTs from the SG library and only one from the FB library. This is a highly significant difference, indicating that this enzyme may be important in chromatin organization associated with tissue specific expression in tsetse SG [124].

#### Transcription factors and transcription machinery

Two hundred and thirty five clusters coding for putative transcription factors (TF) were found in the salivary gland transcriptome of *G. m. morsitans*, one of which is significantly up regulated in the SG library, GM-1732, and assembled from 13 ESTs originating exclusively from the SG library. GM-1732 codes for a homolog of the *Drosophila *product named *salivary gland-expressed bHLH *gene, also known a *sage*, which is a salivary gland specific transcript of the product which interacts with Forkhead (Fkh) TF to block salivary gland apoptosis in *Drosophila*. Abrams *et al*, 2006 indicated that some of the salivary expressed genes of the Fkh family encode subunits of resident endoplasmic reticulum (ER) enzymes, which hydroxylate prolines in collagen and other related proteins. The loss of these Fkh genes leads to a reduced secretory content in *Drosophila *SG [125]. Interestingly, one member of the Forkhead family, coded by GM-2687, is also found expressed in the salivary glands of *Glossina*. Among the salivary expressed TF, we also highlight XBP-1, coded by GM-754 involved in chaperone expression in the ER [126], and the GATA transcription factor [127]; additionally, three transcripts found exclusively in the SG transcriptome code for the homologs of *Drosophila pasilla *isoform I and K. The *pasilla *gene has been identified as expressed and required in *Drosophila *salivary gland and are similar to the human Nova-1 and Nova-2, which are nuclear RNA-binding proteins and directly regulate mRNA splicing. In particular, the human homologs are required for correct splicing of the inhibitory GABA(A) receptor gamma 2 subunit, as well as a glycine receptor subunit [128]. *Pasilla Drosophila *mutants have abnormal salivary glands and have defects in apical secretion [129]. Additional file 2 presents 43 full length and one truncated protein sequences that are putative transcription factors. Transcripts coding for enzymes involved in RNA interference (RNAi) were also found, such as Argonaute and the *Drosophila *homolog of *Aubergine*, which is another PIWI-motif-containing protein and part of the RNAi Dicer machinery. Additional file 2 presents the sequence of 95 proteins, mostly full length, that are putatively associated with the transcription machinery.

#### Signal transduction

Several transcripts were found coding for a diversity of G coupled protein receptors similar to those described previously for binding N-methyl-D-aspartate (NMDA) and glutamate, biogenic amines, including serotonin (5-HT) and peptides, as well as the pre-synaptic latrophilin-like receptor. These receptors may be associated with regulating salivary gland secretion. Receptor subunits for NMDA/glutamate were also found before in *An. gambiae *salivary transcriptomes [18], but they were never studied, to our knowledge, in the context of saliva output regulation in insects, although NMDA affects ink gland synthesis and release from cuttlefish [130]. Peptide neurohormone innervation has been identified previously in the salivary glands of cockroaches [131] and grasshoppers [132], and it may also regulate salivation in tsetse. Serotonin is known to innervate and regulate salivary secretion in diverse insects such as the cockroach [133] and anopheline mosquitoes [134], or to stimulate salivation in calliphorid flies where they were shown over 20 years ago by Michael Berridge to stimulate the inositol phosphate pathway, found later to be widespread in animals [135,136]. The inositol phosphate pathway may be important also in tsetse, indicated by the finding of transcripts associated with several specific inositol kinases and phosphatases. Transcripts coding for nitric oxide synthase (discussed above) and intracellular guanylate cyclase indicate an active NOS signaling pathway in the salivary glands. Additional file 2 describes 194 protein sequences putatively associated with signal transduction pathways, some of which are involved in protein export or immune signaling. Several agonist hormone receptors are also included, mostly as protein fragments.

#### Transporters and channels

Transcripts coding for various vacuolar ATPase (V-ATPases) subunits are shown in additional file 1. V-ATPases are ubiquitous in eukaryotes where they drive electromotive power across cellular membranes that can be used to pump various ions and water depending on the ion selective channels in the particular membrane [137]. They were shown to be activated by 5-HT in *Calliphora *salivary glands [138,139], thereby inducing a K^+ ^rich salivary secretion. Many transcripts were also found coding for various water channels of the aquaporin family and the alpha-1 subunit of the Ca^++ ^channel, the voltage gated Ca^++ ^channel, the rectifier potassium channel, the tandem pore K^+ ^channel, Cl^- ^and ligand gated ion channels. Transcripts were also found coding for cell membrane Na^+ ^+ K^+ ^ATPase subunits and the Ca^++ ^dependent ATPase of the sarcoplasmic reticulum. Sixty four mostly full length protein sequences in this category are included in additional file 2.

#### Cytoskeletal proteins

A somewhat odd result arising from the differential abundance of transcripts among the 3 different libraries is the finding of significantly increased transcription of cytoskeletal proteins of the myosin and actin family in the SG library, with seven clusters of these types of transcripts being significantly up regulated in the SG (additional file 1, worksheet Sg-Upregulated). This may indicate the importance of secretory vesicle transport machinery, or the existence of a subset of these genes specifically associated with SG function.

### Transposable elements (TE)

Transposable element-derived transcripts have been regularly found in salivary transcriptomes of insects and ticks [17,18,45,140], where they may indicate either active transposition, or more probably, suppression of element transposition in the organism's genome. Both Class I and Class II transposable element products are expressed, including type I polyproteins and reverse transcriptases, and type II transposases similar to piggyBac and Mariner. Many of these transcripts show stop codons and frame shifts, indicating they may function as negative regulators of transposition. However, a truncated transcript coding for 307 amino acids of a piggyBac transposase indicates recent insertion in the *G. m. morsitans *genome. Additional file 2 includes 11 TE associated protein sequences.

### Viral sequences (M)

Salivary glands of *G. m. morsitans *and *G. pallidipes *are known to contain viruses [141-143]. Twenty nine ESTs grouped in 12 clusters, found exclusively in the salivary gland library, match viral proteins encoded by at least two different viruses, one of which had its genome recently sequenced [34,36]. Eleven of the clusters match this viral genome, but the most abundant cluster with 17 transcripts, codes for a capsid protein of a picorna like virus similar to deformed wing viruses of insects. This transcript has no homology to the recently described envelop protein of the *Glossina pallidipes *salivary gland hypertrophy virus [144]. The abundant expression of this capsid protein in the salivary glands (and not in fat body or midgut) of *Glossina *suggests that this virus may actually be transmitted from fly to fly via the salivary glands, and accordingly must involve the vertebrate host in this life cycle. If this is the case, either the vertebrate host may amplify this virus, or perhaps infection occurs by co-feeding flies in the absence of a vertebrate host viremia. On the other hand, the artificial co-feeding of large number of flies on a single pool of blood may have created conditions for spread of this virus in the colonized fly, as can occur with co-feeding ticks on a non-parasitemic host [145,146].

### How divergent are salivary proteins?

**Figure 6:**
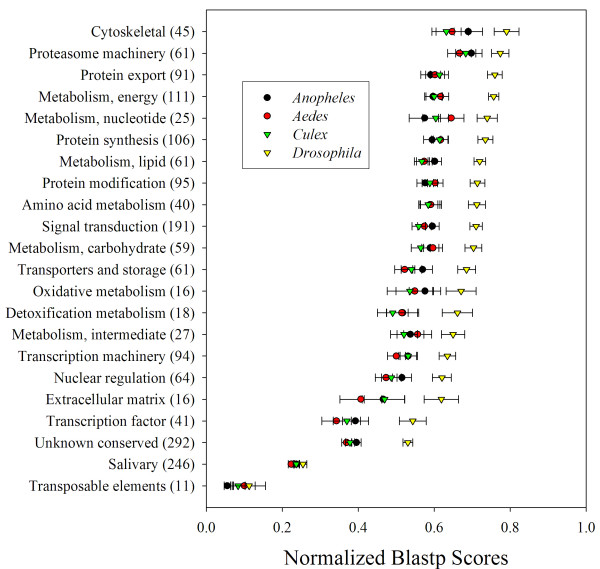
**Normalized blast scores of *Glossina morsitans morsitans *proteins compared to *Drosophila melanogaster*, and three species of mosquitoes: *Anopheles gambiae, Aedes aegypti *and *Culex quinquefasciatus***. Symbols and bars represent the average and standard error of the mean. The number in parenthesis indicates the number of sequences compared for each functional classification.

It is apparent from the deducted CDS (additional file 2) that the putative salivary gland proteins are often most distantly related to other known proteins, when compared by BLASTp to the GenBank NR database, confirming previous suggestions that the salivary proteins of blood sucking arthropods are rapidly evolving [9]. For further comparisons we utilized the blast score ratio approach [147] to compare the *Glossina *proteins to those of other Diptera. A subset of the functionally annotated CDS found expressed in the salivary glands was blasted using BLASTp against the proteomes of *Drosophila melanogaster *and the mosquitoes *Anopheles gambiae, Aedes aegypti *and *Culex quinquefasciatus *(This subset and analysis can be verified in worksheet named 2Analyze-2 of Additional file 2, Table S2). Each best matching score was divided by the score obtained when the *Glossina *proteins was blasted against itself, to obtain the normalized blast score (NBS), which accordingly can vary from 0 to 1. This set excluded *Glossina *proteins that were related at equal or higher than 85% similarity, to exclude bias of the SG group that contains many alleles or closely related gene products. Results indicate that *Drosophila *proteins have overall the highest normalized scores when compared with tsetse proteins (as expected from the phylogeny) and that salivary proteins have the lowest NBS of the whole group, except for the exogenous transposable elements group (Figure 6). When the NBS of the SG set is compared with the combined non SG set shown in Figure 6, their values are highly significantly different from each other (P < 0.0001) for each of the four species (Kruskall-Wallis Anova). This result supports the idea that salivary gland genes of unrelated haematophagous arthropods were acquired in a scenario of convergent evolution, and the differences between proteins possibly magnified by fast evolution due to the host immune pressure on the protein products [9].

### Preliminary characterization of the salivary proteome of *Glossina morsitans morsitans*

**Figure 7:**
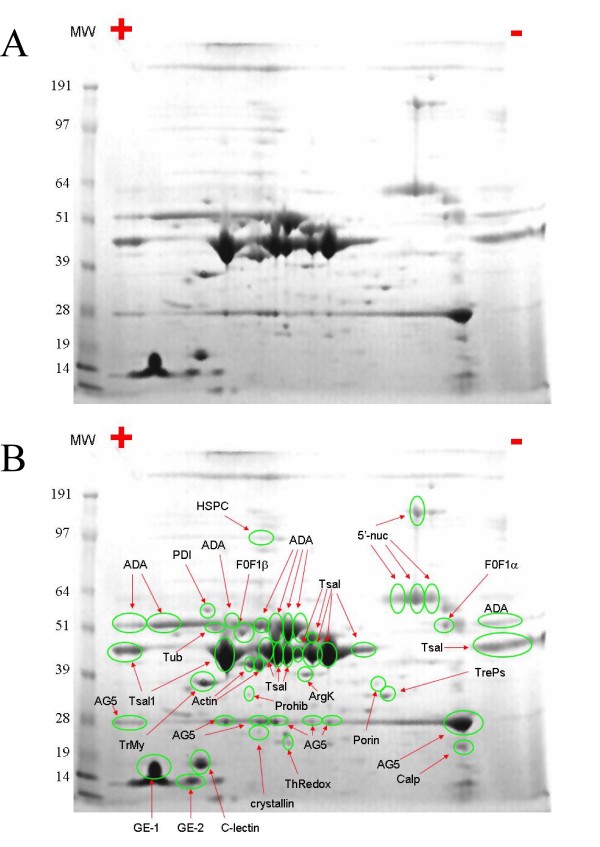
**2D gel electrophoresis of *Glossina morsitans morsitans *salivary gland homogenates**. Numbers on the left indicate the molecular mass marker positions in the gel. The + and - symbols indicate the anode or cathode side of the isoelectrophocusing first dimension, which incorporates a pI range of 3.0 - 10.0. (A) Unmarked gel. (B) Protein spots identified (following tryptic digestion and mass spectrometry) are labeled on the gel. In some cases, several spots were identified as the same protein. For experimental details, see Materials and Methods.

To obtain information on protein expression in the salivary glands of *G. m. morsitans*, we performed a two dimensional (2D) gel electrophoresis separation of the salivary gland homogenate followed by proteolytic digest of the cored blue stained bands (Figure 7) and subsequent tandem mass spectrometry (MS/MS) on the tryptic peptides. Additional file 1 and S2 shows the matching sequence hits obtained by MS/MS. In many cases, due to the similarities between protein members of the same family, it was not possible to assign a unique hit to a particular protein, however, it did indicate the presence of a particular protein family in a gel spot. A detailed description of the findings is given below:

### Endonuclease/Tsal family

Inspection of the gel (Figure 7A) shows a concentration of major protein spots indicative of cationic to neutral products situated between of 40-50 kDa. Nine such spots were identified as diverse members of the endonuclease/Tsal family (Labeled Tsal in Figure 7B). In agreement with the location in the gel, the Tsal1 family has predicted mature masses ranging from 42-43 kDa and acidic isoelectric points varying from 4.8-6.6. Nine distinct spots for this protein are not suprising as we previously proposed that a minimum of eight genes likely encoded this protein family in *G. m. morsitans*. However, it is highly probable that some of the bands also represent post-translational modifications. The abundance of this protein family suggests a main function for this family may reside not in an enzymatic capacity, but rather in sequestration of inflammatory or hemostasis agonists, including immunoglobulin binding. Indeed, the most abundant secreted proteins found in mosquitoes, ticks and triatomine bugs are involved in binding and sequestration of biogenic amines such as histamine, serotonin norepinephrine or adenosine nucleotides [102,148-150]. The high protein concentrations are needed because these proteins can bind one or two agonist molecules at most, and they need to produce at least one μM protein concentration at the site of the bite to neutralize these agonists (see discussion of this subject in [102]).

### Adenosine deaminase family

Eight gel spots with molecular weigh retention near the 51 kDa marker had mass spectral hits with members of the ADA family of enzymes, which have predicted mature molecular masses between 40-54 kDa, and isoelectric points varying from highly acidic (<4.0 - 5.5) to an extremely basic (>9.0) group. These are marked ADA in Figure 7B. Over 55% sequence coverage by MS/MS was achieved for gi|5817644 and 63% coverage for GM-1228. These eight gel spots are in agreement with the proposed minimum of seven genes coding for ADA enzymes expressed in the salivary glands of *G. m. morsitans*.

### 5' nucleotidase family

Four gel spots produced matches to gi|14488055 and its allele GM-784, most abundantly at the basic end of the gel, running as several spots at ~64 kDa and one much higher spot at ~150 kDa. These spots are marked 5'-nuc in Figure 7B. The protein sequences with MS hits have predicted mature masses of 59.4 kDa in accordance with the location on the gel, and pI of 7. The latter is not in accordance with the protein location on the gel, thus suggesting either a different gene, or an eroneous pI prediction due to non-standard ionization of charged amino acids, or post translational modifications that would create a more basic protein. The higher molecular form may represent an insoluble dimer, as this has previously been reported for the apyrase of *Triatoma infestans *[151]. The relatively high expression of this enzyme in *Glossina *salivary glands is paralleled by similarly high expression of apyrase members of the 5'nucleotidase in mosquito sialoproteomes [17,152-154], and accounts for the highest apyrase specific activities found in any other secretory organ thus far measured [155]. It remains to be determined, however, whether *Glossina *apyrase activity arises from a 5' nucleotidase family member.

### Antigen 5 family

The intense basic spot, observed at 28 kDa, and gradually stretches across the entire gel, had peptide sequence match to members of the antigen-5 family, which have predicted mature molecular weight of 27.1-22.8 (labeled as AG5 on Figure 7B). All four members of this family described in additional file 2 have basic pI's of 8.2-8.6, which matches the location and intensity of the major spot, but does not match the remaining acidic trail. These bands could again be the produce of either different genes or from post-translation modifications (eg, phosphate additions). Indeed, multiple phosphorylation sites for protein kinase C, casein and tyrosine kinases exist in all related proteins annotated in additional file 2. However, it could also be simply a result of protein overloading, which would cause a comet effect as the ISO gel reached the point of saturation.

### GE family

An intense band is also observed at an acidic region of the gel running at ~14 kDa. This band and a nearby, less intense band of smaller molecular mass, correspond to the Gly-Glu rich proteins uniquely found in *Glossina*, annotated as GM-2815 (GE-1 on Figure 7B) and GM-3689 (GE-2 on Figure 7B) in additional file 2.

### C type lectin

A distinct acidic gel spot, running at ~18 kDa, was identified as a C-type lectin named GM-591 (three MS fragment hits) and GM-595 (two MS fragment hits) in additional file 2. The spot is labeled as C-lectin in Figure 7B. This spot is probably represented by sequence GM-591, which has a predicted pI of 4.9 and mass of 17.7 kDa, as opposed to GM-595 that has a predicted pI of 6.9 and mass of 12.5 kDa.

### Housekeeping products

The putative housekeeping gene products for a heat shock protein cognate (HSPC), the chaperone crystallin, a thioredoxin peroxidase (ThRedox), the alpha and beta subunits of the F0F1 ATPase, arginine kinase (ArgK), two actin spots, tropomyosin (TrMy), calponin (Calp), prohibitin (Prohib), and trehalose-6-phosphate synthase (TrePs) were also unequivocally identified and labeled accordingly in Figure 7B.

### Toward the characterization of the sialoimmunome of Glossina morsitans morsitans

**Figure 8:**
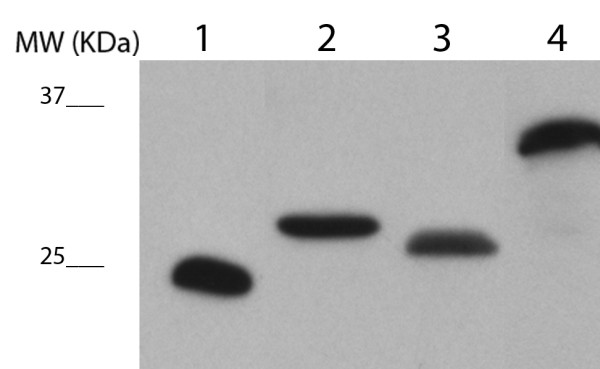
**Western blot analysis of four recombinant proteins found in tsetse saliva**. Purified His-tagged peptides were resolved using a 12.5% SDS-PAGE gel and subjected to Western blot using an anti-tsetse saliva antiserum. The order of peptides loaded on the gel is: His_6_Tag-5 (positive control, lane 1), His_6_-GMsg-06 h03 (2), His_6_-GMsg-15f12 (3), and His_6_-GMsg-45 g06 (4). Molecular weights from Protein Markers are indicated on the left.

Aiming to identify immunogenic secreted saliva proteins in our tsetse salivary gland EST database, we targeted 20 proteins, including both predicted secreted and housekeeping (control) proteins (Table 4) for recombinant expression. We then used a rabbit anti-*Glossina morsitans morsitans*-saliva antiserum to immunodetect these recombinant proteins using standard Western blotting (WB) techniques. This protein expression set included a truncated variant of the previously characterized tsetse TAg-5 protein [27,156], also expressed as a His-tagged peptide (His6-TAg5) to be used as positive control. His6-TAg5 was strongly recognized by the anti-saliva antiserum (data not shown) and was used as positive control in all subsequent WB experiments. All 19 recombinant proteins produced the projected molecular weights (MW) when resolved with SDS-PAGE stained with SeeBlue (data not shown). Out of the 20 peptides expressed, only four were recognized by the anti-saliva antiserum (Figure 8): His¬6-GMsg-15f12 (lane 3), His_6_-GMsg-45 g06 (lane 4), and His6-GMsg-06 h03 (lane 2), and the control, His6-TAg5 (lane 1). However, when compared to the His6-TAg5 control, the other protein bands were considerably weaker (data not shown). These weaker immunoblot signals for the other three proteins could simply be due to a lower protein-specific antibody titre in the polyclonal antiserum, thus reflecting either the natural abundance of those proteins in the saliva or the reduced protein immunogenicity of these proteins when compared to Tag5 (in lieu of accepting ineffective protein expression). Alternatively, it was plausible that *Glossina *TAg5 could act as an immunoglobulin binder as recently described for the homologous salivary protein from *Stomoxys calcitrans *[95], which would inadvertently amplify the signal and bias results. Control experiments were done to assess whether *Glossina *TAg5 could bind non-immune serum or secondary antibody alone; the negative cross-reaction with control antibodies confirmed that tsetse TAg5 does not share this Ig-binding characteristic with the stable fly homologue (results not shown). The three additional polypeptides recognized by the anti-saliva antiserum were two enzymes (a phopholipase A2 and a serine protease) and a mucin. This infers that these proteins (or closely related family members) may be secreted in saliva, and may be epidemiological markers of tsetse exposure as observed with ticks [157-161], mosquitoes [162-165] and sand flies [166-169].

**Table 4:** ESTs expressed as His6-tagged proteins and screened with anti-saliva antiserum

	EST Cluster/singleton	Equivalent cluster on S1 datasheet	Template DNA	Size (KDa)*	Western-Blot	Best match to deducted orfs
1	TAg-5-t	sgmgfb-contig_369	GMsg-41d10	24	**positive**	Tsetse TAg-5
2	GMsg-7018	sgmgfb-contig_4047	GMsg-15f12	26.9	**positive**	secreted salivary phospholipase A2, group XIIA
3	GMsg-8079	sgmgfb-contig_5222	GMsg-45 g06	35.45	**positive**	salivary mucin
4	GMsg-7305	sgmgfb-contig_1606	GMsg-06 h03	28.8	**positive**	Salivary expressed serine protease
5	GMsg-4676	sgmgfb-contig_4626	GMsg-116b04	18.6	negative	conserved hypothetical protein
6	GMsg-4602	sgmgfb-contig_16226	GMsg-76e12	29	negative	Synaptotagmin
7	GMsg-4579	sgmgfb-contig_16256	GMsg-77 g03	10	negative	Serine proteinase inhibitor (KU family)
8	GMsg-7037	sgmgfb-contig_5650	GMsg-149 h01	47	negative	Molecular chaperone (DnaJ superfamily)
9	GMsg-7847	sgmgfb-contig_6058	GMsg-164 h01	37	negative	ATP synthase, subunit b
10	GMsg-8221	sgmgfb-contig_2150	GMsg-116b02	24	negative	Unknow
11	GMsg-9862	sgmgfb-contig_14095	GMsg-123a01	19	negative	Putative arsenite-translocating ATPase
12	GMsg-10713	sgmgfb-contig_15840	GMsg-58e12	26	negative	Xaa-Pro aminopeptidase
13	GMsg-3464	sgmgfb-contig_315	GMsg-06 h09	21,5	negative	Ferritin-1 heavy chain
14	GMsg-5226	sgmgfb-contig_1969	GMsg-58 g10	43	negative	Peptidase A1
15	GMsg-6756	sgmgfb-contig_1231	GMsg-169a09	32	negative	Unknown
16	GMsg-6848	sgmgfb-contig_984	GMsg-94e07	18.6	negative	Unknown
17	GMsg-8002	sgmgfb-contig_699	GMsg-102d06	35.5	negative	Unknown
18	GMsg-5890	sgmgfb-contig_290	GMsg-148f11	22	negative	Serpin
19	GMsg-8367	sgmgfb-contig_930	GMsg-115a09	58.7	negative	Serine-type endopeptidase
20	GMsg-8399	sgmgfb-contig_499	GMsg-17 g08	56	negative	disulfide isomerase

## Conclusions

The sialotranscriptome of tsetse reported here is made unique among other haematophagous arthropods analyzed thus far by the sheer number of ESTs collated (over 20,000, others being under 2,000 ESTs), by the normalization protocol used (all other libraries were not normalized), and by its assembly with other large tissue transcriptomes, namely fat body and midgut, thus permitting the identification of differential tissue expression. Although the sialotranscriptomes originating from distinct organisms not sharing a common haematophagous ancestor are very different from each other, a common pattern is emerging, including the following generic classes: (1) enzymes, (2) protease inhibitors, (3) agonist chelators (named kratagonist in Ribeiro and Arca, [170]) (4) antigen 5-related proteins, (5) mucins, (6) immunity related products and (7) the unexpected. Additionally, it is also found that haematophagous saliva contains many proteins deriving from multigenic families.

The *G. m. morsitans *salivary repertoire follows this pattern, as detailed in additional file 2. The *Glossina *sialome reveals alleles of previously described gene products, that previously known salivary proteins are multigenic, identifies at least 7 new multigenic polypeptide families unique to *Glossina*, and additionally lists over one hundred possible secreted peptides. Most of these proteins have no known function and provide a discovery platform for identification of novel pharmacologically active compounds, novel vector-based vaccine targets, and immunological markers of vector exposure.

## Methods

### Materials

Standard laboratory chemicals were purchased from Sigma Chemicals (St. Louis, MO) if not specified otherwise. Formic acid and trifluoroacetic acid (TFA) were obtained from Fluka (Milwaukee, WI). Trypsin was purchased from Promega (Madison, WI). HPLC-grade acetonitrile was from EM Science (Darmstadt, Germany) and water was purified by a Barnstead Nanopure system (Dubuque, IA).

### Biological material

The *G. m. morsitans *colony maintained in the insectary at Yale University was originally established in Bristol from puparia from fly populations in Zimbabwe. Flies are maintained at 24 ± 1°C with 50-55% relative humidity, and receive defibrinated bovine blood every 48 h using an artificial membrane system [171].

### Normalized EST library construction and sequencing

For library preparation approximately 800 pairs of salivary glands were microscopically dissected from male and female adult flies that were two weeks old, 48 hours post their last blood meal. A quantity of more than two μg of total RNA was extracted using TRIzol^®^Reagent (Invitrogen, Carlsbad, CA, USA) according to manufacturer's instructions. mRNA was selected using the PolyATtract^® ^mRNA Isolation System (Promega, Madison, WI, USA). For library construction, first strand cDNA synthesis was primed with a *Not*I-tag-oligo-(dT)18. The tag is a sequence of ten nucleotides that is unique for this library and thus serves as an identifier. The resulting DNA/RNA hybrid was treated with RNase H and then used as a template for DNA Poll-catalysed second-strand synthesis. After the addition of *Eco*RI adaptors, the double-stranded cDNAs were digested with *Not*I and size-selected. The resulting molecules were directionally cloned into the *Eco*RI and *Not*I sites of the phagemid vector, pT7T3PAC. The library then went through one round of normalization performed according to 'method 4' [172,173]. This procedure is based on the hybridization of PCR-amplified cDNA inserts of a library with the library itself in the form of single-stranded circles. Following hybridization to a relatively low Cot of 5-10, the remaining single-stranded circles (normalized library) are purified over hydroxyapatite (HAP), converted to double-stranded circles by primer extension and electroporated into bacteria [172]. Each clone was sequenced using a T3 or T7 primer using ABITM big dye terminator kits.

### Bioinformatic tools and procedures used

Expressed sequence tags (ESTs) were trimmed of primer and vector sequences, clustered, and compared with other databases as described [153]. The BLAST tool [174] and the CAP3 assembler [175] were used to assemble the database, as well as to compare it to other databases and pipe the results into a hyperlinked Excel spreadsheet, as described in the dCAS software tool [31]. ClustalW [176] and Treeview software [177] were used to align sequences and visualise alignments. Phylogenetic analysis and statistical Neighbor Joining (NJ) bootstrap tests of the phylogenies were done with the Mega package [178]. For functional annotation of the transcripts we used the tool BlastX [179] to compare the nucleotide sequences to the NR protein database of the National Center for Biotechnology Information (NCBI) and to the Gene Ontology (GO) database[180]. The tool RPSBlast [179] was used to search for conserved protein domains in the Pfam [181], SMART [182], Kog [183] and Conserved Domains Databases (CDD) [184]. We have also compared the transcripts with other subsets of mitochondrial and rRNA nucleotide sequences downloaded from NCBI, and to several organism proteomes downloaded from NCBI (yeast), Flybase (*Drosophila melanogaster*), or ENSEMBL (*An. gambiae*). Segments of the three-frame translations of the EST (because the libraries were unidirectional we did not use six-frame translations), starting with a methionine found in the first 100 predicted AA, or to the predicted protein translation in the case of complete coding sequences, were submitted to the SignalP server [185] to help identify translation products that could be secreted. O-glycosylation sites on the proteins were predicted with the program NetOGlyc [105]. Functional annotation of the transcripts was based on all the comparisons above. Mass spectrometry results were mapped to the excel spreadsheets using a home made program. The following example illustrates the convention for interpreting the data: The hit MS-31-3 -> TPTAELR 111| represents the result of the third peptide (MS-31-**3**) identified in the tryptic digest from spot number 31 (MS-**31**-3), which produced the sequence TPTAELR that start at position 111 of the protein to which it is a hit, and is on the first cell of the particular spreadsheet row.

Comparisons of deducted coding sequences with the proteome of *D. melanogaster, Aedes aegypti, Anopheles gambiae *and *Culex quinquefasciatus *(Downloaded from Flybase or VectorBase) where done by the normalized score obtained from Blastp comparisons. The reference score was created by obtaining the score of the *Glossina *protein sequence blasted against itself, as proposed for the Blast Score Ratio approach [147].

When attempting identification of multi gene families, we attributed transcripts coding for proteins that were more than 10% different in their primary amino acid sequence to derive from different genes.

### 2D Gel Electrophoresis, tryptic digestion-MS/MS experiment

2D gel electrophoresis was performed using the ZOOM IPGRunner System (Invitrogen) under manufacturer's recommended running conditions. Briefly, approximately 130 μg of sample proteins were solubilized with 155 μl rehydration buffer (7 M urea, 2 M thiourea, 2% CHAPS, 20 mM DTT, 0.5% carrier ampholytes, pH 3-10). The samples were absorbed by rehydration ZOOM strips (7 cm; pH 3-10 NL) overnight at room temperature and then focused under manufacturer's recommended conditions. The focused IPG strips were reduced/alkylated/equilibrated with reducing and then alkylation reagents dissolved in the sample buffer. The strips were then applied onto NuPAGE 4-12% Bis Tris ZOOM gels (Invitrogen). The gels were run under MOPS buffer and stained with SeeBlue staining solution (Bio Rad). A total of 60 spots were selected for tryptic digestion, based on their staining intensity. Protein identification of 2D gel separated proteins was performed on reduced and alkylated trypsin digested samples prepared by standard mass spectrometry protocols. Tryptic digests were analyzed by coupling the Nanomate (Advion BioSciences)--an automated chip based nano electrospray interface source--to a quadrupole time of flight mass spectrometer, QStarXL MS/MS System (Applied Biosystems/Sciex). Computer controlled data dependent automated switching to MS/MS provided peptide sequence information. AnalystQS software (Applied Biosystems/Sciex) was used for data acquisition. Data processing and databank searching were performed with Mascot software (Matrix Science). The NR protein database from the NCBI, National Library of Medicine, NIH, was used for the search analysis, as was a protein database generated during the course of this work.

### Expression of recombinant proteins

Twenty peptides (Table 4) were expressed as His-tagged proteins. These peptide sequences were predicted from the consensus sequence of selected clusters available from GeneDB and GenBank (Table 4), which had a signal peptide indicative of secretion [185]. DNA minipreps corresponding to their sequences were used as template to amplify the coding sequence (excluding signal peptide sequences) from each putative protein. A list of specific primers sequences used can be provided upon request. His-tagged proteins were expressed as His-6-peptides from a pET28a vector (kindly provided by Prof. Peter A. Williams from University of Wales, Bangor) in BL21(DE3)pLysS strain, and purified using HisBind Quick 300 Cartridges (Novagen, Cambridge, UK) following the manufactures' instructions.

### Western Blot analysis using rabbit anti-Glossina morsitans saliva polyclonal antiserum

The polyclonal antiserum used was as reported before [27]. Briefly, a total of 25 pairs of salivary glands were dissected from 15-day-old male flies and collected in ice-cold PBS. The outflow fluid (= tsetse saliva), containing approximately 100 μg saliva proteins, was emulsified in Freund's complete adjuvant and subcutaneously injected in a New Zealand white rabbit. Two subsequent boosters of the same antigen preparation in Freund's incomplete adjuvant were administered at 3-week intervals. The polyclonal anti-saliva serum was collected 2 weeks after the final booster. For the Western Blot experiments, purified peptides were diluted 1:2 in 2× loading buffer (4% SDS, 20% Glycerol, 0.2% Bromophenol Blue, 200 mM DTT, 100 mM Tris-Cl, pH 6.8) and resolved using a 12.5% SDS-PAGE gel. Peptides were then transferred to Hybond-P PVDF membranes (Amersham Biosciences UK Limited, Bucks, UK), at 90 V for 1 hour, blocked for 40 minutes in blocking solution (5% fat-free milk in PBT) and probed overnight at 4°C with the anti-tsetse saliva rabbit anti-serum [27] diluted 1:7,500. After incubation with primary antiserum, the membranes were washed in PBT (3 × 10 minutes) and probed at room temperature for one hour with a conjugated goat anti-rabbit IgG- HRPO secondary antiserum (Pierce Biotechnology, Rockford, USA; dilution: 1:50,000). SuperSignal West Dura Extended Duration Substrate (Pierce Biotechnology, Rockford, USA) and Kodak BioMax MR film were used for Western Blot development.

## Authors' contributions

BSM constructed the normalized library for subsequent sequencing. GA participated in the bioinformatic analysis and contributed to writing the draft manuscript. JdS performed the Western blot analysis and contributed to the writing of the draft. JMCR has performed bioinformatic analysis and wrote the bulk of the manuscript. LH helped with the Western blot analysis. JvdA provided the anti-saliva antiserum and helped to design the study. MB, MJL and SA helped to conceive, design and co-ordinate the study and assisted with the writing of the draft. ZH dissected salivary glands and prepared the mRNA. All authors have read and approved the final manuscript.

## Supplementary Material

Additional file 1 Hyperlinked Microsoft Excel file with assembled EST's and various database comparisons. Click here for file

Additional file 2 Hyperlinked Microsoft Excel file with coding sequences and various database comparisons. Click here for file
